# Epithelial splicing regulatory protein 1 and 2 paralogues correlate with splice signatures and favorable outcome in human colorectal cancer

**DOI:** 10.18632/oncotarget.12070

**Published:** 2016-09-16

**Authors:** Abigail J. Deloria, Doris Höflmayer, Philip Kienzl, Justyna Łopatecka, Sandra Sampl, Martin Klimpfinger, Tamara Braunschmid, Fabienne Bastian, Lingeng Lu, Brigitte Marian, Stefan Stättner, Klaus Holzmann

**Affiliations:** ^1^ Division of Cancer Research, Department of Medicine I, Comprehensive Cancer Center, Medical University Vienna, Austria; ^2^ Department of Pathology and Bacteriology, Social Medical Center South, Kaiser Franz Josef Hospital, Vienna, Austria; ^3^ Department of Surgery, Social Medical Center South, Kaiser Franz Josef Hospital, Vienna, Austria; ^4^ Department of Chronic Disease Epidemiology, Yale School of Public Health, School of Medicine, Yale Cancer Center, Yale University, New Haven, USA; ^5^ Department of Visceral, Transplantation and Thoracic Surgery, Innsbruck, Austria; ^6^ Institute of Pathology, University Medical Center Hamburg-Eppendorf, Hamburg, Germany

**Keywords:** colorectal cancer, epithelial splicing regulatory protein, microsatellite instability, overall survival, prognostic marker

## Abstract

ESRPs are master splice regulators implicated in alternative mRNA splicing programs important for epithelial-mesenchymal transition (EMT) and tumor progression. ESRP1 was identified in some tumors as good or worse predictor of outcome, but in colorectal cancer (CRC) the prognostic value of ESRPs and relation with mesenchymal splice variants is not clear. Here, we studied 68 CRC cases, compared tissue expression of ESRPs with clinical data and with EMT gene splice patterns of conditional CRC cells with deficient ESRP1 expression.

Around 72% of patients showed global decreased transcript expression of both ESRPs in tumor as compared to matched non-neoplastic colorectal epithelium. Reduction of ESRP1 in tumor cells was evaluated by immunohistochemistry, associated with microsatellite stability and switch to mesenchymal splice signatures of FGFRs, CD44, ENAH and CTNND1(p120-catenin). Expression of ESRPs was significantly associated with favorable overall survival (log-rank test, *P*=0.0186 and 0.0408), better than prognostic stratification by tumor staging; and for ESRP1 confirmed with second TCGA cohort (log-rank test, *P*=0.0435). Prognostic value is independent of the pathological stage and microsatellite instability (ESRP1: HR=0.36, 95%CI 0.15–0.91, *P*=0.032; ESRP2: HR=0.23, 95%CI 0.08–0.65, *P*=0.006).

Our study supports the role of ESRP1 as tumor suppressor and strongly suggests that ESRPs are candidate markers for early detection, diagnosis, and prognosis of CRC.

## INTRODUCTION

Colorectal cancer (CRC) is the third most common cancer in men and second in women worldwide [1]. Thus, it is a pressing need to broaden the knowledge about genes involved in this disease. Several gene families of growth factor receptors are associated with cancer development including fibroblast growth factor receptors (FGFRs) [2]. FGFR1-4 signaling pathways are activated upon FGF ligand binding and regulate important biological processes such as tissue development, regeneration, angiogenesis, and cancer. Hence, FGFRs are discussed as putative therapeutic targets [2–5]. FGFRs are highly subjected to alternative splicing, especially its Ig-like III domain with IIIb and IIIc variants observed in FGFR1-3 [6, 7]. This domain plays a critical role as it determines ligand binding specificity. Furthermore, the IIIb and IIIc variants are tissue-specific, such that the former is preferentially expressed in epithelial and the latter in mesenchymal cells. However, in CRC the FGFR3 IIIc variant was found to increase predominantly in a subgroup of advanced tumors and to exert oncogenic functions by a gain of broader ligand specificity important for tumor progression [8]. Moreover, FGFR2 IIIc variant was recently identified to drive EMT in epithelial cells [9].

Epithelial splicing regulatory proteins (ESRPs)1 and 2 have been identified as key regulators for Ig-like III domain variant splicing of FGFR2 [10]. ESRPs are epithelial-specific RNA binding proteins which promote splicing of epithelial FGFR2 IIIb variant and transcript variants from genes associated with epithelial-mesenchymal transition (EMT) such as CD44, ENAH, CTNND1 (p120-catenin). Moreover, ESRPs function as mastermind splice regulators providing an additional post-transcriptional layer of gene regulation by hundreds of alternative splicing events that contributes to shape the EMT process in tumors [11, 12]. However, alternative splicing events of FGFR1 and 3 by ESRPs are not yet well understood.

Isoform switching of FGFR and EMT are regulated by exogenous signaling mediated by TGF-beta as recognized in mouse normal mammary epithelial cells [13]. Microarray-based analyses demonstrated in this murine cell model that TGF-beta dependent transcription factors induce broad alteration of splicing patterns by downregulating ESRPs [14]. Mutation of ESRP genes is another possibility for deregulation of these genes in cancer in addition to epigenetics [15] and EMT [16]. Frame-shift mutation in a specific exon of ESRP1 was identified in CRC cell lines with microsatellite instability (MSI) using gene identification by NMD inhibition (GINI) assay [17]. The mutation causes rapid degradation of the mutated transcript and was identified in around 50% of primary colon tumors with MSI but not in colon tumor cell lines with microsatellite stability. In breast cancer patients ESRP1 was associated with lower patient survival rate [16], in contrast to pancreatic ductal adenocarcinoma where increased ESRP1 expression was related to better survival [18].

Here, we studied a CRC patient cohort and evaluated the correlation of ESRPs with EMT gene variant splicing and clinical data such as MSI and overall survival. Epithelial splice patterns were compared between cell models with conditional ESRP1 expression and CRC tumor tissues.

## RESULTS

### ESRPs expression in CRC

ESRPs transcript expression profiles were studied by Genevestigator in more than 300 anatomical human parts and indicate highest expression in colon tissue and lowest expression in fibroblasts and immune cells [19]. This first result suggests good usability to explore ESRPs as candidate prognostic markers for CRC.

**Figure 1:**
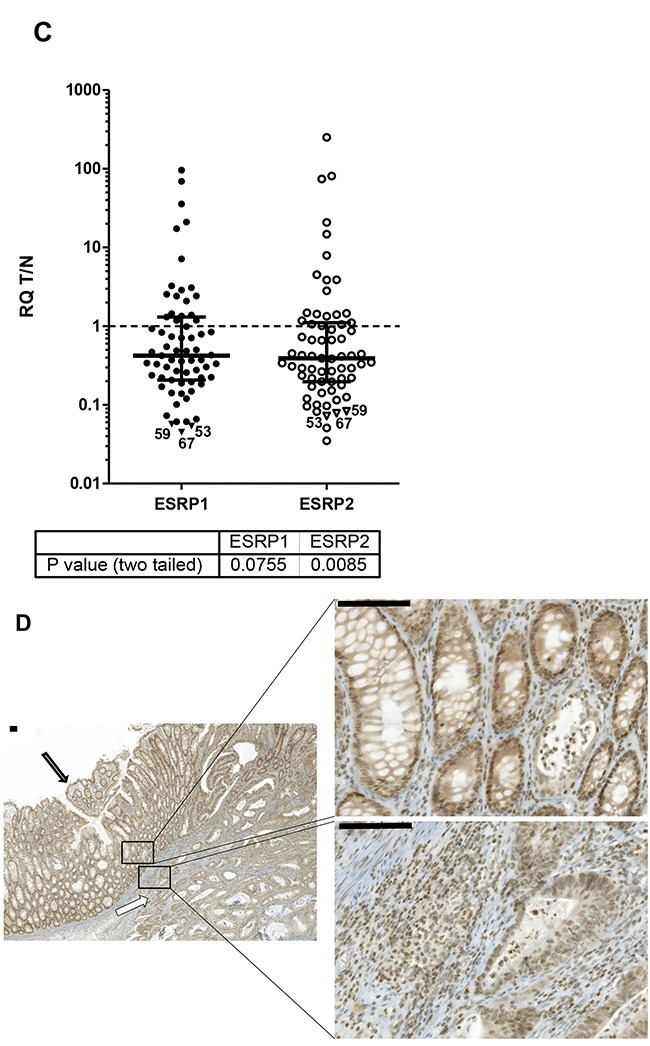
ESRP1 and ESRP2 expression in CRC **A.** ESRP1 (closed circles) and ESRP2 (open circles) transcript expression in relation to ribosomal 36B4 as reference gene and to a reference tissue shown as scatter plot of all CRC cases. Relative quantity (RQ) levels for matched T and N tissue are different by P values <0.0001 (***) tested by Wilcoxon signed rank test. Median RQ values of T and N for ESRP1 were 82.6 and 163, and for ESRP2 142 and 354. Median with interquartile range is indicated by error bars. **B.** ESRP1 and ESRP2 expression correlate in T and N CRC tissues. Relative quantity (RQ) levels for T and N CRC tissues were log transformed to yield normal distribution of data and analyzed for linear correlation with Pearson method (P values of <0.0001). Linear regression line with 95% Confidence Intervals (dashed) and Pearson correlation coefficients R square are indicated. **C.** Ratios of RQ levels for matched T and N tissue (T/N) of transcript expression are shown as scatter plot. Wilcoxon signed rank test against theoretical median of 1 resulted P values of 0.076 and 0.009 for ESRP1 and ESPR2, respectively. Cases studied by IHC with lowest ESRP1 values are indicated by triangles and numbers. Median with interquartile range is indicated by error bars. **D.** Representative IHC of CRC cases with minor transcript expression in tumor tissue case #59 showed in normal colon epithelium cells (closed arrow) a stronger ESRP1 expression in the nuclei compared to adjacent invasive carcinoma cells (open arrow). Bars indicate 100μm.

Tumor (T) and adjacent non-tumor (N) tissue from the resection border from patients diagnosed with CRC was studied by qPCR for relative expression of ESRP1 and ESRP2 (Figure 1). From the analyzed 68 patient cohort two cases differ in such that one case (#35) had no matching non-tumor tissue and another case (#67) was a non- invasive tubulovillous adenoma with focal high-grade dysplasia (Table 1). Median RQ transcript levels for ESRP1 and ESRP2 decreased significantly 0.51- and 0.40-fold in T compared to N tissue, respectively (Figure 1A). Furthermore, both RQ levels for ESRP1 and ESRP2 correlate high and very high positive in T and N tissue, respectively (Figure 1B, Supplementary Table S1). The identified strong correlation between expression of ESRP1 and ESRP2 support the concept of a general coregulation in colon, both in tumor and non-tumor tissue cells.

**Table 1:** Clinical-pathological data of colorectal cancer patients.*

Number of patients	n	68
Year of surgery	Range	2000-2010
Age (yr)	Median (Range)	70 (26 - 87)
Gender		
	Female, n (%)	26 (38)
	Male, n (%)	42 (62)
Site		
	Colon, n (%)	55 (81)
	Rectum, n (%)	13 (19)
Clinical follow-up available	n (%)	68 (100)
Follow-up time (mo)	Median	62.2
Survival time (mo)	Median	>160
Outcome		
	Favorable, n	48
	Deceased, n	20
UICC stage		
	n.a., n (%)^#^	1 (2)
	I, n (%)	11 (16)
	II, n (%)	28 (41)
	III, n (%)	21 (31)
	IV, n (%)	7 (10)
WHO grade		
	n.a., n (%)^#^	1 (2)
	2, n (%)	45 (66)
	3, n (%)	22 (32)
MSI status		
	Stable, n (%)	57 (84)
	Unstable, n (%)	11 (16)
Tissue	tumor (T), n	68
	non-tumor (N), n	67
mRNA RQ T<N		
	ESRP1, n (%)	48 of 67 (72)
	ESRP2, n (%)	47 of 67 (70)

* cases (n=4) had two synchronous colorectal carcinomas;

# case (n=1) with focal high-grade dysplasia in a tubulovillous adenoma with CRC stage and grade not available (n.a)

For 67 cases the ratios of RQ levels from the matched T and N tissue were plotted (Figure 1C). Up to 72% of cases from the studied patient cohort demonstrated less ESRP1 and ESPR2 expression in T compared to paired N tissue (Table 1). ESRP1 analyzed by IHC confirmed uniform protein expression in tumor cells as e.g. shown for CRC case #59 (Figure 1D). Furthermore, ESRP1 was found to be more strongly expressed in the nucleus of normal colon epithelium cells compared to adjacent invasive carcinoma cells of 2/3rds of the tumor tissues with low transcript expression in T compared to N tissue (Figure 1C). This finding supports the concept of global decreased expression at transcriptional level of ESRPs in tumor cells of the majority of CRC cases.

### Relation of ESRPs and epithelial/mesenchymal splicing patterns in CRC

**Figure 2:**
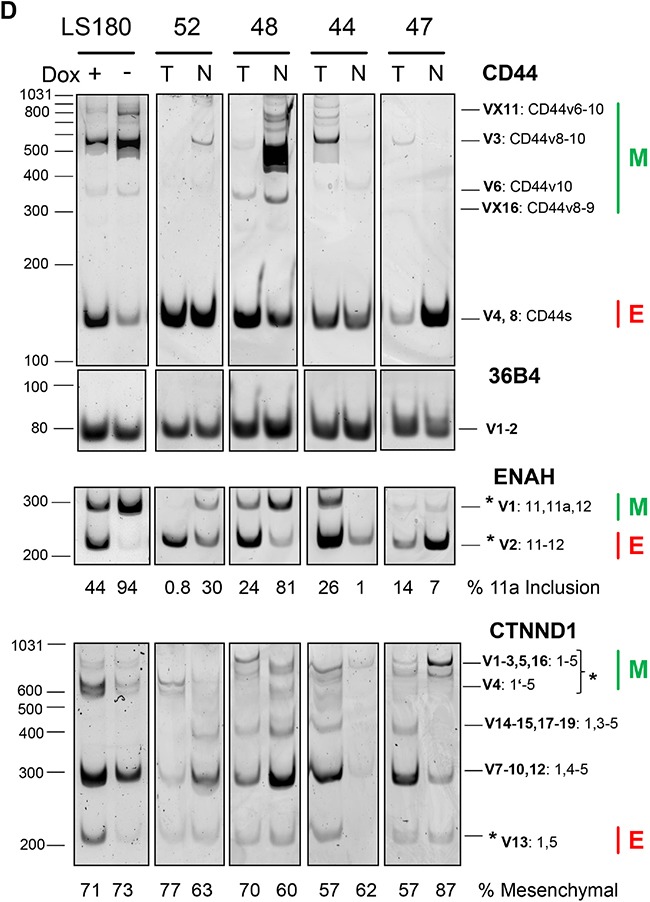
ESRP1 dependent splice patterns in CRC **A, B.** ESRP1 and ESRP2 protein and transcript expression in CRC cell models. (A) SW480 wt and subline mt, and (B) conditional Tet-off LS180-RBM35A cells cultivated 48 hours with (+Dox) and without (−Dox) doxycycline analyzed by Western blotting (left panel) and qPCR (right panel). Beta actin protein expression served as control. Bars for RQ values indicate mean with SEM. **C.** FGFR 1-3 IIIb/IIIc splicing analyses by qPCR of cells with conditional ESRP1 expression (mt or treated with Dox) and of tissue from CRC cases with lower (RQ T/N <1) or higher (RQ T/N>1) expression of ESRP1 in T compared to N tissue. Representative cases were selected from Figure 1C. Dashed line indicates RQ values of control experiments set as 1 for SW480 (wt), for LS180 (cells without Dox), and for CRC cases (N tissue). Bars for RQ values indicate mean with SEM. **D.** CD44, ENAH and CTNND1 (p120-catenin) splicing analyses of conditional Tet-off LS180-RBM35A cells (Dox +/−) and of CRC case (matched T and N tissue) with different expression level of ESRP1 in T compared to N tissue. Results of semi-quantitative PCR and PAGE from indicated genes including reference (36B4) are shown. For each gene assay the PCR cycles have been optimized to allow quantification within linear range. Epithelial (E) and mesenchymal (M) splice variants are indicated by their molecular amplicon size in bp (see Supplementary Table S3) as predicted from reference transcript variants from database. Molecular size from marker is indicated on the left of each representative PAGE analyses. CD44 transcript variants (V) contain standard (s) and variable variants exons (v). Intensity between marked splice variants (asterisks) is given below of each representative PAGE analyses in % for the longer variants of ENAH and CTNND1. CTNND1 V4 resulted by alternative exon 1 usage missing 74 bp as described [44].

We next studied splicing pattern regulation in SW480 and LS180 CRC in vitro cell models with conditional ESRP1 expression and in CRC tissue cases (Figure 2). The SW480 (wt) and a spontaneous derived SW480 subline (mt) showed extreme diminished ESRP1, but not ESRP2 expression (Figure 2A). This subline compared to the mother cell line demonstrated change in cell morphology and accelerated growth rate (not shown). LS180 with homozygous deletion mutation in the ESRP1 gene was engineered to conditionally overexpress recombinant ESRP1. Addition of doxycycline (Dox) for 2 days to LS180 cells reduced ESRP1 transcript and protein expression, but low ESRP2 transcript levels were not affected (Figure 2B). In conditional CRC cells and in selected CRC cases the RQ transcript levels of FGFR1-3 IIIb/IIIc splice variants were analyzed by qPCR (Figure 2C). RQ expression values of SW480 mt cells with low ESRP1 expression were compared to values of wt cells with high ESRP1 expression and showed a decrease in IIIb/IIIc variant ratio for FGFR1-3. Similar, expression values of LS180 cells with low ESRP1 expression (+Dox) were compared to values of cells with high ESRP1 expression (−Dox) and showed a decrease in the IIIb/IIIc variant ratio for FGFR2 and FGFR3, but not for FGFR1. CRC cases with lower but not higher expression of ESRPs in T compared to N tissue showed a similar strong decrease in the splice variant ratio of FGFR2, like in the LS180 cell model with inducible ESRP1 expression. Results indicate that reduced expression of ESRP1 in CRC switches FGFR2 expression to more mesenchymal splice variants with strong potential of disease progression. Further mesenchymal and epithelial splicing events were analyzed from genes known as regulated by ESRP1 and important for tumor progression (Figure 2D). Mesenchymal CD44v splice variants and mesenchymal Exon 11a inclusion of ENAH were reduced in conditional LS180 cells without ESRP1 expression (+Dox) and in T tissue of CRC cases only if ESRP1 was expressed at a lower level than in the matched N tissue. Mesenchymal variants of CTNND1 were affected in case of absence of ESRP1 in tissue of CRC cases, but not in the conditional CRC cell model. Results indicate that ESRP1 regulate in CRC multiple mesenchymal specific gene transcript splice variants important for tumor progression.

Next, RQ levels for FGFR1-3 IIIb and IIIc transcript variants were determined by qPCR in T and N tissue of at least available 40% CRC cases and compared with RQ levels for ESRP1 and ESRP2 (Supplementary Table S1). ESRP1 and ESRP2 demonstrate a very similar pattern of significant correlations with the levels of FGFR1-3 splice variants. RQ transcript levels of ESRP1 and ESRP2 correlate positively with the splice variant ratio IIIb/IIIc of FGFR1 and FGFR2 in N, but no correlation was evident in T tissue. Remarkably, RQs of ESRP1 and ESRP2 in N tissue did not correlate with the ratio IIIb/IIIc of FGFR3 in N but had a negative correlation in T tissue. Results show no significant overall correlations between FGFR1-3 IIIb/IIIc variants and expression of ESRPs in the studied CRC tumor tissue.

### Relation of ESRP1 and ESRP2 expression with clinical and diagnostic data

**Figure 3:**
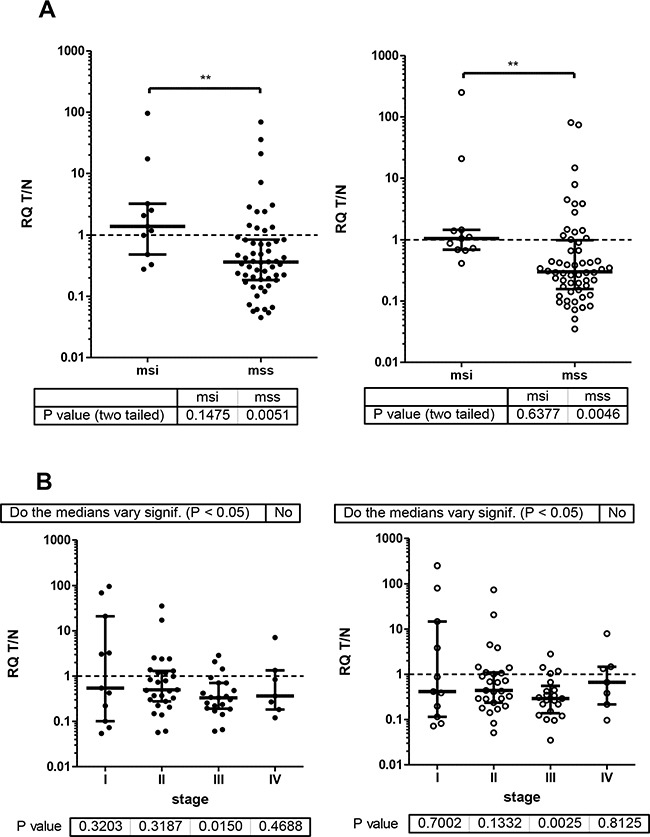
ESRP RQ expression as T/N ratios of CRC patients were grouped by micro-satellite status and tumor stage ESRP1and ESRP2 closed and open circles, respectively. Median with interquartile range is indicated by error bars. Wilcoxon signed rank test of actual median against theoretical median of 1 resulted P values for groups as indicated below graph. **A.** msi and mss groups analyzed by Mann Whitney test resulted P values 0.05-0.001 (**). **B.** Tumor stage I-IV groups analyzed by Kruskal-Wallis test with Dunn's multiple comparison test resulted no significant difference of the medians between tumor stages.

Ratios of RQ levels from the matched T and N tissue demonstrate reduced ESRP1 and ESRP2 expression in most but not all of CRC cases (Figure 1C). The cases were grouped according to clinical-pathological data (Table 1, Figure 3, Supplementary Figure S1). In general, 16% (11 of 68) of cases with MSI do not show reduced ESRP1 and ESPR2 expression in T compared to N tissue (Figure 3A). No such difference between groups was evident when cases were clustered by tumor stage (Figure 3B), tumor grade, tumor site, gender, or age at diagnoses (Supplementary Figure S1). However, if groups were tested against equal expression in T and N tissue (T/N=1), only male cases with MSS, tumor stage III, colon localization and patients below age of 60 showed significant reduction of ESRPs in tumor as compared to normal tissue.

### Relation of ESRP1 and ESRP2 expression with patient outcome

**Figure 4:**
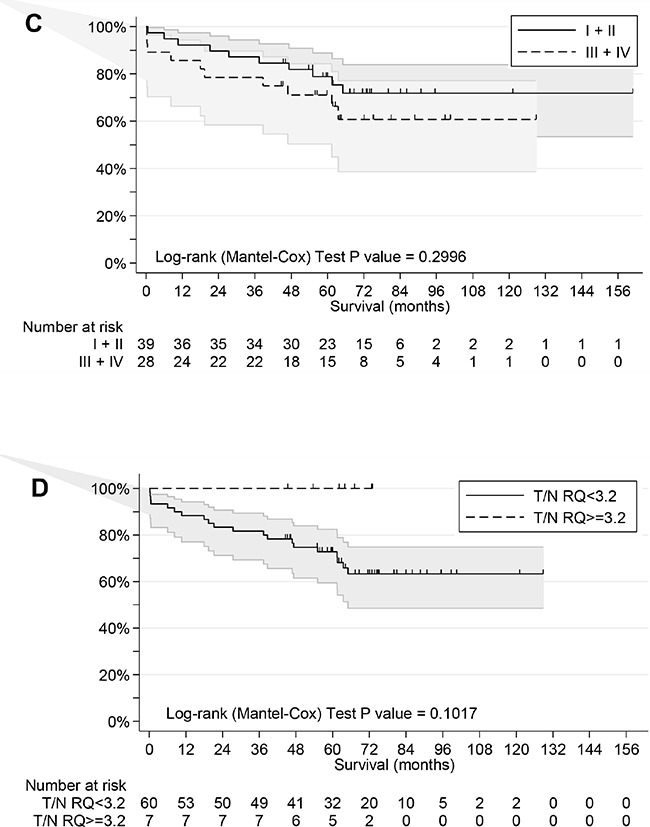
Overall survival analyses of CRC patients **A.** Patients grouped by low and high RQ ESRP1 level (cutoff value 70.4) in T tissue. **B.** Patients grouped by low and high RQ ESRP2 level (cutoff median value) in T tissue. **C.** Patients grouped by low (I+II) and high (III+IV) stages. **D.** Patients grouped by extreme high RQ ESRP1 T/N ratio level (cutoff value 3.2).

Outcome information was available as overall survival (OS) for all patients with median follow-up of 68 months, median survival time of more than 160 months and 48 favorable outcomes (Table 1). Univariate analyses of age, gender, tumor site, stage, grade, MSI status, and expression of ESRPs in T and N tissue was performed (Table 2). Age (*P*=0.068) and high ESRP1 and ESRP2 expression in T (*P*=0.009 and *P*=0.006, respectively) but not in N (*P*=0.217 and *P*=0.304, respectively) tissue were significantly associated with survival. For expression of ESRPs dichotomization was done by values close to the median for better fit of the survival model. Kaplan-Meier survival curves considering these cutoffs are shown (Figure 4). High expression of ESRP1 and ESRP2 in T tissue is associated with favorable OS (Log-rank test, P values 0.0186 and 0.0408) (Figure 4AB). This difference for ESRP1 and ESRP2 corresponded to a hazard ratio (HR) = 0.30 (95% CI 0.12 - 0.74) and 0.26 (95% CI 0.10 - 0.69), respectively (Table 2). These associations were more relevant as observed if cases were grouped into low (I, II) and high (III, IV) stages (Log-rank test, *P*=0.2996) (Figure 4C). In contrast, no similar association of outcome was observed with RQ T/N ratios (Table 2). However, cases with extreme high ESRP1 RQ T/N ratios (>=3.2) were associated with favorable overall survival (Log-rank test, *P*=0.1017) (Figure 4D).

**Table 2:** Cox regression model

	n				Multivariate****		
		Univariate			with T, N and T/N		
		HR	95% CI	p	HR	95% CI	p
**ESRP1_T**							
<66.67	25	ref			ref		
>=66.67	43	0.30	0.12 - 0.74	**0.009**	0.36	0.15 - 0.91	**0.032**
**ESRP1_N**							
<66.67	17	ref			ref		
>=66.67	51	0.56	0.22 - 1.41	0.217	0.38	0.12 - 1.15	**0.087**
**ESRP1_T/N**							
<0.269	22	ref			ref		
>=0.269	45	0.47	0.19 - 1.14	**0.094**	0.43	0.15 - 1.22	0.113
**ESRP2_T**							
<131.1	29	ref			ref		
>=131.1	39	0.26	0.10 - 0.69	**0.006**	0.23	0.08 - 0.65	**0.006**
**ESRP2_N**							
<131.1	18	ref			ref		
>=131.1	50	0.62	0.25 - 1.55	0.304	0.56	0.18 - 1.72	0.309
**ESRP2_T/N**							
<0.668	41	ref			ref		
>=0.668	26	2.11	0.87 - 5.09	**0.097**	2.10	0.76-5.80	0.153
**Instability**							
Instable	11	ref					
stable	57	1.04	0.30 - 3.54	0.954			
**Gender**							
f	26	ref					
m	42	1.29	0.51 −3.23	0.589			
**Tumor site**							
rectum	13	ref					
colon	55	0.67	0.24-1.85	0.443			
**Grade**							
2	45	ref					
3	22	1.21	0.48 - 3.04	0.685			
**Stage**							
I	11	ref					
II	28	0.85	0.22 - 3.29	0.815			
III-IV	29	1.39	0.38 - 5.00	0.629			
p-trend		1.28	0.67 - 2.44	0.455			
**Age**		1.05	1.00 - 1.10	**0.068**			

**** adjusted with age, stage, grade, tumor site, gender, microsatellite stability for each gene expression in each type of tissue in bold are marked p values <0.1.

For multivariate analysis age, stage, grade, tumor site, microsatellite status, gender, and expression of ESRPs were retained in the final regression model (Table 2). Prognostic values of ESRPs in T tissue were maintained after adjusting for all other covariates, supporting an independent role as biomarkers for prediction of OS of studied patient cohort. The HR for patients with high ESRPs was reduced to 23-36% (ESRP1: HR=0.36, 95% CI 0.15–0.91, *P*=0.032; ESRP2: HR=0.23, 95% CI 0.08–0.65, *P*=0.006). The concordance index values for ESRP1 and ESRP2 with other multivariate variables in T tissue were AUC=0.74, 95% CI 0.61-0.87, *P*=0.0004 and AUC=0.76, 95% CI: 0.63-0.89, *P*<0.0001, and suggest good overall prognostic accuracy of the final model.

For validation of ESRPs as prognostic markers we used as a second independent cohort the public TCGA COADREAD dataset with samples analyzed for gene expression of CRC patients with follow-up information available (n=411). Expression levels of ESRPs were standardized and patients were grouped in tertian. Kaplan-Meier survival curves of patients with low, mean, and high ESRP expression were compared (Supplementary Figure S2). Low compared to mean and high expression of ESRP1 resulted shorter OS (Log-rank test, *P*=0.0435). In detail, median OS of such patients was reduced around 50%, from 100 (95% CI: 49 - >140) and 93 (95% CI: 61 - >140) months for mean and high ESRP1 expression, respectively, to 56 (95% CI: 45 - 100) months.

Multivariate analysis were adjusted for patients age, gender, stage, and microsatellite status, and demonstrated for low compared to mean and high ESRP1 expression in tumor a borderline significance with HR=0.56, 95% CI 0.31-1.01, *P*=0.055 and HR=0.62, 95% CI 0.36-1.09, *P*=0.098. In contrast, ESRP2 resulted no significant association with OS (Log-rank test, *P*=0.123).

## DISCUSSION

This study investigates ESRP1 and 2 expression as well as alternative splicing patterns in paired tumor and non-tumor tissue of CRC patients and their correlation with clinical data. The results demonstrate a co-expression of both paralogs and indicate that expression of ESRPs in tumor is associated with a prolonged overall survival. In contrast, CRC cells *in vivo* and *in vitro* with reduced expression showed splicing patterns associated with EMT. Our results strengthen a role of ESRPs as tumor suppressors with strong prognostic capacity for patients with CRC by monitoring of key alternative splicing programs important for tumor progression.

Most of CRC cases studied (~70%) demonstrated reduced expression of both ESRP1 and ESRP2 in tumor cells compared to adjacent non-tumor tissue. The observed co-regulation in tumor tissue might mirror that both ESRPs are downregulated during EMT and both suppress cancer cell motility through distinct mechanisms [20]. The same authors report plasticity of ESRP expression during squamous cell carcinogenesis. ESRPs became down-regulated in invasive fronts, but were re-expressed in neoplastic cells in the lymph nodes, where tumor cells metastasize and colonize. Such plastic expression also might be important for early colon carcinogenesis as we observed reduced ESRP1 expression in the single carcinoma in situ case #67 of a non-invasive tubulovillous adenoma. In adenomas and carcinomas of the colon decreased expression of ESRPs might indicate the presence of EMT and thus disease progression.

The CRC cell models with conditional ESRP1 expression demonstrated a similar splicing regulatory program in EMT as in CRC tumor and adjacent non-tumor cells. In detail, 2 to 3 FGFRs decreased the ratio of IIIb/IIIc splice variants in the conditional cell models. FGFR1 was not regulated in LS180 compared to SW480 cells indicating a tumor cell type dependent mechanism of IIIb/IIIc splice regulation by ESRP1. However, in human CRC cases with reduced ESRP1 expression, only the FGFR2 IIIb/IIIc splice variant ratio is diminished. Similar correlation between low ESRP1, low IIIb, and high IIIc variant expression for FGFR2 was demonstrated by IHC in pancreatic cancer [18]. FGFR2 and FGFR3 are generally expressed with a more minor expression of FGFR1, in a panel of screened CRC cell lines (unpublished data). However, broad indications for relevance of all FGFRs in CRC already exist [6]. In contrast to FGFR2 IIIb, the IIIc variant expression correlated with distant metastasis and poor prognosis, and with aggressive behavior important for tumor progression *in vitro* and *in vivo* [21, 22]. FGFR2 is part of the recently identified CRC molecular landscape of clinically actionable kinase targets [9]. FGFR2 promises potential as biomarker and therapeutic target in other cancer subtypes. E.g. the study of RNA sequencing data of clear cell renal cell carcinomas identified for FGFR2 transcripts a switch from IIIb to IIIc variant as associated with worse clinical features like higher grade and shorter survival [23]. We recently demonstrated for FGFR3 IIIc oncogenic functions by a gain of broader ligand specificity important for CRC tumor progression [8]. Our data now support the existence of CRC subgroups with decrease in ESRP expression but increase of mesenchymal splice variants in multiple FGFRs (at least FGFR2 and 3) important for tumor progression.

The cell surface protein CD44 modulates cellular signaling cascades important for tumor progression [24]. CD44 is a major marker for stem-like cancer cells and is highly expressed in metastatic cancer cells [25]. Our recent work confirmed that CD44-positive colorectal adenoma cell growth and survival depend on autocrine FGF18/FGFR3-IIIc signaling, thus indicating importance of this pathway already in precursor lesions of colon cancer [26]. Our result of decreased CD44v splice variants in CRC tumor cells are supported by findings in metastatic breast cancer cells that changed the expression from CD44v to CD44s variant isoforms on the cell surface after depletion of ESRP1 [16]. In breast cancer cells this CD44 isoform switch led to reduced stability of the cytosine transporter xCT important for metastasis to the lung and to suppression of lung colonization. Furthermore, favorable overall survival of breast cancer patients was associated with low ESRP1 expression. In contrast, survival of CRC patients of this study was associated with high expression of ESRPs in primary tumors, potentially reflecting diverse expression in time and function during tumor progression of cancer subtypes. E.g. recent work in primary melanoma demonstrated that high levels of CD44v6 splice variant correlate with expression of ESRPs and predict development of melanoma brain metastasis [27]. Overexpression of ENAH in the invasive front of CRC was already identified and suspected to have a role in the initial steps of tumor invasion from primary sites [28]. We demonstrated with the studied patient cohort and the conditional cell model that expression of ESRP1 in CRC tumor cells is associated with a shift in EMT splicing patterns of ENAH and CTNND1 (p120-catenin) [10], thus confirming a key role of ESRPs in CRC progression. These master splicing regulatory proteins are coordinators of a complex alternative splicing network that adds an important post-transcriptional layer on gene expression important for disease progression [11, 29]. However, we identified correlations between ESRPs and FGFR1-3 IIIb/IIIc splice variants in non-neoplastic colorectal epithelium but not in the matched adjacent tumor. Absence of correlation between the expression of ESRPs and FGFR splice variants in T tissue support the concept of heterogeneity and common deregulation of splicing factors in tumor cells [30]. Another more simple explanation of the absence of correlation would be the different degree of contamination of tumor tissue samples with fibroblasts with high expression of FGFR genes. It has to be mentioned that ESRPs itself may exist not only in full-length, but also in truncated forms. E.g. the majority of melanoma highly express only the last four exons 13-16 that presumably do not generate ESRP1 protein with normal function [29]. This truncated ESRP1 transcript variant was found apparently melanocytic specific and the presence in other types of cancer including colon was rare. Primers used in our study for qPCR detection of ESRP1 are located on exon 1 and 2, and include the start codon of the gene. Thus, our assay detects all transcript variants that use the regular ESRP1 start codon and does not identify variants that exist at other positions like the variant from melanoma.

Expression profiles of ESRPs in CRC were compared with all clinical-pathological categories studied. Identified subgroups with decreased T/N ratios <1 represent patients with a high potential for metastatic diseases progression and worse outcome. Only cases with MSI showed no decrease in expression of ESRPs in the tumor tissue compared to adjacent non-neoplastic colorectal epithelium. MSI is found in 15-20% of colorectal cancer which is in line with 16% of MSI in our CRC cohort [31]. However, our finding is in contrast to the high incidence (11 of 23) of primary colon tumors with MSI that include a specific frame-shift mutation in the coding region of the ESRP1 gene [32]. This specific mutation in ESRP1 cause degradation of mutated transcripts by a mechanism termed nonsense-mediated decay [17, 32]. Such a mutation is expected to significantly reduce ESRP1 expression in around 50% of CRC cases with MSI. These published data do not explain the identified positive correlation of ESRP1 expression with MSI in the patient cohort studied and therefor remains to be further investigated.

MSI patients from our cohort did not demonstrate better OS compared to MSS which is in agreement as recently reported [33]. However, studies identified also a more favorable outcome for MSI patients compared to MSS [31]. Tumors of MSI patients harbor defects in DNA mismatch repair genes that result mutations in genes like ESRP1 and TGFBRII [17]. Such mutations caused by DNA strand slippage generate novel frameshift peptides within the tumor with highly immunogenic and inflammatory potential. This anti-tumor response was suggested as a model to control hereditary nonpolyposis CRC or Lynch Syndrome, which develops over the life-time of an individual, from an autosomal dominant mutation in at least one of the DNA mismatch repair genes [34]. Our finding of higher ESRP1 expression in tumors of MSI compared to MSS patients does not support such a model. In contrast, our data showed that high ESRP1 as well as ESRP2 in tumor tissue are associated with favorable overall survival outcome. The association of ESRP1 was confirmed by a second independent CRC patient cohort available from TCGA data. Moreover, regression models identified ESRPs as potential independent prognostic biomarkers, but need validation by further studies. The positive association of ESRP expression with favorable prognosis in the studied CRC patient cohort is supported by the identification of ESRP1 as tumor suppressor [32]. In detail, the authors used ESRP1-null LS180 CRC cells (with MSI status) engineered to conditionally regulate expression of ectopic ESRP1 and identified for ESRP1 deficient cells grown in vitro a change in morphology with reduced adhesion and increased anchorage independent growth and grown *in vivo* as xenografts in nude mice an increase of tumor volume. Our data now reports identical splicing patterns between this conditional cell model and tumor tissue of CRC patients. We observed identical growth characteristics to the tumor cells reported above with the spontaneous derived SW480 CRC subline (with MSS status) deficient for ESRP1 expression (unpublished data).

In summary, ESRPs in CRC cells may prevent, independent of MSI, the expression of multiple mesenchymal gene splice variants involved in tumor progression. ESRPs as master splice regulators for EMT may be less elaborate but similar effective as scoring of EMT by transcriptomics to study its dynamics in cancer progression, treatment response, and survival [35]. Such EMT scores were associated with poorer survival in ovarian and colorectal, but not breast carcinomas. Similarly, ESRPs correlated in our CRC study with favorable outcome. This is in agreement to pancreatic ductal adenocarcinoma where increased ESRP1 expression was related to better survival [18], but in contrast to breast cancer patients where ESRP1 was associated with lower survival rate [16]. Recent findings in melanoma patients identified a link between low ESRP1 expression in tumor tissue and tumor-associated immune cytolytic activity with better patient survival [29]. Whether ESRPs play a positive and/or negative role during cancer progression in specific tumors must be elucidated on a tumor-by-tumor basis and considering homogeneity of tumor tissue.

## MATERIALS AND METHODS

### Patients, tissue and cell lines

The study population consisted of 68 patients diagnosed with colorectal cancer (CRC), who had undergone resection at Kaiser Franz Josef (KFJ) Hospital (Table 1). Tumor (T) and from the resection border matched adjacent non-neoplastic colorectal epithelium as non-tumor (N) tissue samples were snap frozen in liquid nitrogen immediately after surgical removal and stored at −80°C until use. Demographic, surgical, and pathological data were collected in a prospectively maintained database at KFJ. Clinical follow-up was retrospectively collected until end of 2013 for all patients. The study was approved by the local ethics committee. Formalin-fixed/paraffin-embedded (FFPE) blocks with tumor and adjacent non-tumor tissue were available for validation. Overall survival was calculated from the date of the first surgery at which CRC was confirmed histologically and FFPE tumor blocks were stored. Human colorectal cancer cell lines SW480 and LS180 with status MSS and MSI were obtained as CCL-228 and CL-187 from the American Type Culture Collection (ATCC, Manassas, VA). SW480 termed wild-type (wt) and a spontaneous derived outgrown subline termed mutant (mt) with change from epithelial to mesenchymal morphology were cultured with RPMI-1640 (Sigma-Aldrich, St. Louis, USA), clonal purified by serial dilution assays and authenticated by short tandem repeat profiling (Genetic Resources Core Facility, Johns Hopkins Institute of Genetic Medicine, Baltimore, MD). LS180 were obtained from the Ionov Laboratory (Roswell Park Cancer Institute, Buffalo, NY) and cultured with minimal essential medium as recommended. All cell lines were grown with 10% FCS, 1% Penicillin/Streptomycin under standard tissue culture conditions (5% CO2 at 37 °C). The subline Tet-off LS180-RBM35A that includes a conditional expressed hESRP1 was cultured additionally with 150μg/ml hygromycin B (PAA Laboratories, Pasching, Austria), and 2μg/ml doxycycline (Clontech Laboratories, CA, USA) for conditional expression [32].

### MSI status and protein detection

Four μm thick sections from FFPE tissue blocks were subjected to routine analysis with IHC detection kit (Dako) for DNA mismatch-repair (MMR) proteins MLH1 (Novocastra, 1:100), MSH2 (Cellmarque 1:150), MSH6 (Novocastra, 1:200), and PMS2 (BD Biosciences Pharmingen 1:150) [36]. Loss of MMR protein expression was detected by the absence of nuclear staining in tumor cells. Adjacent lymphocytes and normal colon epithelial cells served as internal control. Tumors with loss of expression of at least one of the DNA MMR proteins or only focal positive expression were further analyzed by multiplex PCR using Gene Amp PCR System 9700 (Applied Biosystems) and by capillary electrophoresis using Genetic Analyzer 3500 DX (Applied Biosystems) for stability status of seven microsatellite-loci: BAT-25, BAT-26, Mono-27, NR-21, NR-24, Penta-C, and Penta-D (Ingenetics). For one MSI case #55 different microsatellite loci (D5S346, BAT-26, BAT-25, D2S123) were used. Tumors with expression of all markers detected by IHC were classified as microsatellite stable (MSS) [37]. For ESRP1 detection by IHC, 2 μm thick sections from FFPE tissue blocks were prepared and routinely stained with the automated IHC/ISH slide staining system BenchMark Ultra (Roche). Representative slides were scanned with 40x objective by Panoramic Midi Slide Scanner (3DHISTECH, Budapest, Hungary) and visualized with Panoramic Viewer Software. Anti-RBM35A antibody ab107278 (ESRP1, Abcam) was diluted 1:75. Same primary antibody was diluted 1: 625 to detect ESRP1 in sub-confluent cells by western blotting on polyvinylfluoride membrane (GE Healthcare life sciences Whatmann Westeran) using RIPA buffer with recommended protocol. Anti-ESRP2 antibody ab113486 was diluted 1:1000 (Abcam). Protein samples were measured by Coomassie Protein Assay Dye Reagent Concentrate (Bio-Rad) and 25 μg were subjected to each well of 10% SDS-PAGE. Secondary antibody (Goat anti-Rabbit IgG HRP coupled, Abcam) was diluted 1:1000. Antibody for detection of beta-actin (Sigma-Aldrich, MO, USA) was used for equal load control as recommended by the manufacturer. ChemiDoc Touch Imaging System (Bio-Rad) and X-ray film (Kodak) were used for detection with ECL Prime Western Blotting Detection Reagent (GE Healthcare).

### RNA extraction and RT-qPCR

Total RNA extracts were prepared from tissue and cells as described with minor modifications [38]. In brief, TRIzol reagent (Invitrogen) and ceramic beads with Precellys tissue homogenizer (Peqlab, Germany) were applied. Reverse transcription (RT) was performed with 1 μg total RNA for complementary DNA synthesis using RevertAid First Strand cDNA synthesis kit (Fermentas, Germany) with the RevertAid Premium Reverse Transcriptase at 55°C. Aliquots of cDNA corresponding to 40 ng of RNA and GoTaq qPCR Master Mix (Promega, WI, USA) were applied for quantitative real-time PCR (qPCR) with 200nM of both forward and reverse primers [39]. In brief, transcript levels of ESRP1, ESRP2, FGFR1-3 IIIb and IIIc, and reference gene 36B4 were measured in triplicates. qPCR conditions: 10 min at 95°C, 40 cycles: 15 sec at 95°C, 1 min at 60°C. The relative quantities (RQ) with 95% confidence interval (CI) value were calculated for each gene in respect to expression levels of 36B4 and to the gene expression of another arbitrarily chosen sample on the plate (7500 Software Version 2.0.6, Applied Biosystems, CA, USA). Efficiencies of qPCR assays were taken into account when calculating RQ values. For efficiency rates standard curves were generated with reamplification of diluted qPCR products as the starting point and four consecutive 1:2 dilutions. Primer details and qPCR efficiencies used for RQ calculations are described in Supplementary Table S2 or published [39].

### Semi-quantitative PCR

PCR was setup on a peqSTAR 96X Universal Thermocycler (Peqlab, Germany) in 30μl including 2xGoTaq Hot Start Colorless Master Mix (Promega) with 200 nM primers and aliquots of cDNA corresponding to 75ng total RNA. PCR conditions: 5 min at 95°C, 38 cycles: 30 sec at 95°C, 30 sec at 60°C, and 90 sec at 72°C. After 18, 23, 28, 33, and 38 cycles aliquots of 3 μl were taken out, mixed with loading buffer and analyzed by 5% PAGE with MassRuler Low Range DNA Ladder (Fermentas). DNA was detected after EtBr staining by Typhoon laser scanner for imaging and quantification (GE Healthcare Life Sciences). CD44, ENAH, and CTNND1 primer sequences and the predicted amplicon sizes of alternative splice variants calculated from reference transcripts are described in Supplementary Table S3.

### Statistical analysis

Level of ESRP expression on Affymetrix Human Genom U133 Plus 2.0 Array was analyzed in silico by Genevestigator [19]. Dataset used as clinical validation cohort TCGA_COADREAD_exp_HiSeqV2-2015-02-24 was downloaded from https://genome-cancer.ucsc.edu/ and is combined from TCGA colon (COAD) and rectal adenocarcinoma (READ) datasets that are based upon public data generated by the TCGA Research Network: http://cancergenome.nih.gov/ [40]. Dataset contains patient follow-up information and gene-level transcription estimates from Illumina HiSeq 2000 RNA Sequencing, as in RNA-Seq by Expectation-Maximization (RSEM) normalized count [41]. Computations were performed with GraphPad Prism software version 5.02 (San Diego, California, USA), STATA release 13 (StataCorp LP, College Station, TX, USA) and SPSS version 22.0 (IBM Corp, Armonk, NY, USA). All statistical tests were two sided, and P ≤ 0.05 was considered significant. Correlations among RQ values were assessed after log transformation with the Pearson correlation calculation and linear regression analysis. Mann Whitney U test or paired t-test was used for group comparison as indicated. Kaplan-Meier analysis and the log-rank test were applied to illustrate the relation of survival with parameters of patients. The cutoff values of ESRP1 and ESRP2 expressions in tumors, a point best distinguishing survivors and non-survivors, which was determined by an unsupervised algorithm of the maximization of hazard ratio as described elsewhere [42, 43]. Univariate and multivariate survival analysis was performed using the Cox regression model with exact method to handle ties in SAS program (SAS version 9.4).

## SUPPLEMENTARY MATERIALS TABLES FIGURES




