# Supplementary material for: Epithelial splicing regulatory protein 1 and 2 paralogues correlate with splice signatures and favorable outcome in human colorectal cancer
Source: Oncotarget. 2016 Sep 16;7(45):73800–16. doi: 10.18632/oncotarget.12070 (PMC5342015; doi:10.18632/oncotarget.12070)
Supplement: Supplementary file 1 [file oncotarget-07-73800-s001.pdf]

Epithelial splicing regulatory protein 1 and 2 paralogues correlate with splice signatures and favorable outcome in human colorectal cancer

SUPPLEMENTARY FIGURES AND TABLES

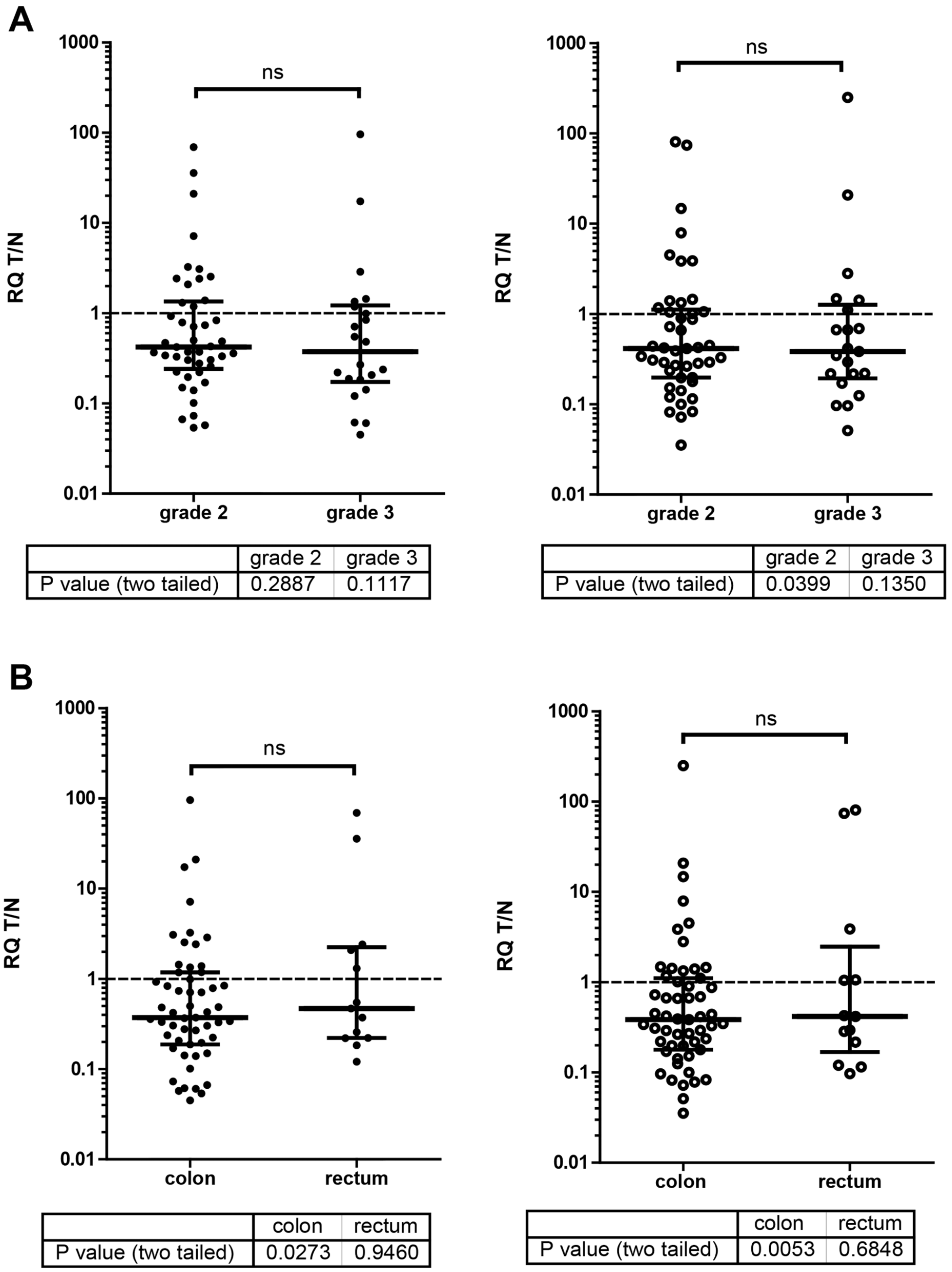

Supplementary Figure S1: ESRP RQ expression as T/N ratios of CRC cases were grouped by tumor grade A. and site B. gender C. and by age at diagnoses D. ESRP1and ESRP2 closed and open circles, respectively. Median with interquartile range is indicated by error bars. Wilcoxon signed rank test of actual median against theoretical median of 1 resulted P values for groups as indicated below graph. A-C. Groups analyzed by Mann Whitney test resulted no significant (ns) P values <0.05. (*Continued*)

C

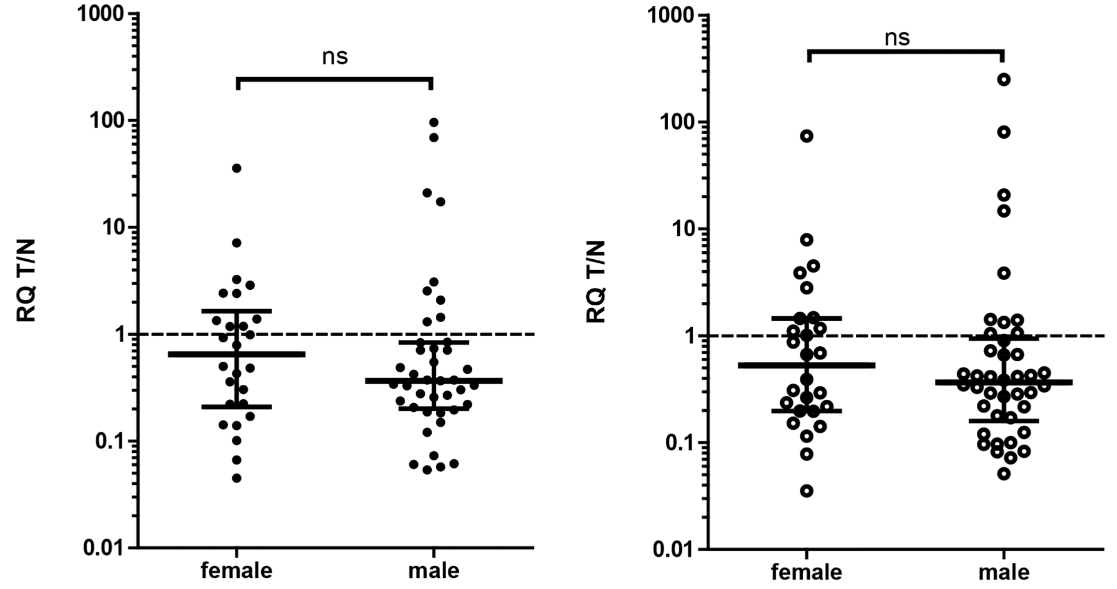

	female	male
P value (two tailed)	0.7222	0.0504

	female	male
P value (two tailed)	0.4020	0.0049

D

Do the medians vary signif. (P < 0.05)	No

Do the medians vary signif. (P < 0.05)	No

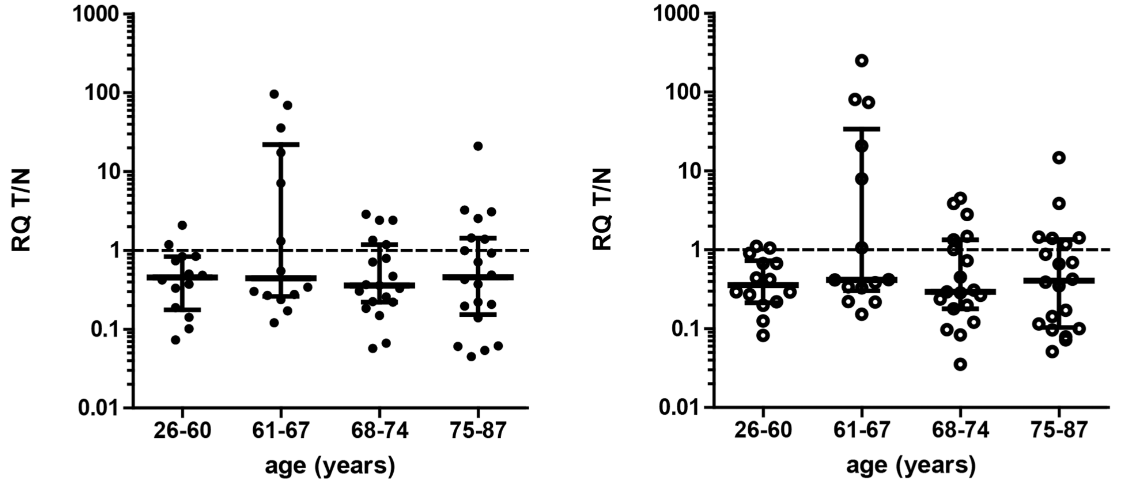

26-60	61-67	68-74	75-87
0.0245	0.6257	0.1531	0.4222

26-60	61-67	68-74	75-87
0.0009	0.6257	0.1909	0.0894

Supplementary Figure S1: (*Continued*) ESRP RQ expression as T/N ratios of CRC cases were grouped by tumor grade A. and site B. gender C. and by age at diagnoses D. ESRP1and ESRP2 closed and open circles, respectively. Median with interquartile range is indicated by error bars. Wilcoxon signed rank test of actual median against theoretical median of 1 resulted P values for groups as indicated below graph. A-C. Groups analyzed by Mann Whitney test resulted no significant (ns) P values <0.05. D. Four age groups analyzed by Kruskal-Wallis test with Dunn’s multiple comparison test resulted no significant vary of the medians between age groups.

A Standardized ESRP1 grouping:
group 0 (-4.800, -0.219, n=139); group 1 (-0.216, 0.531, n=134); group 2 (0.540, 2.47, n=138)

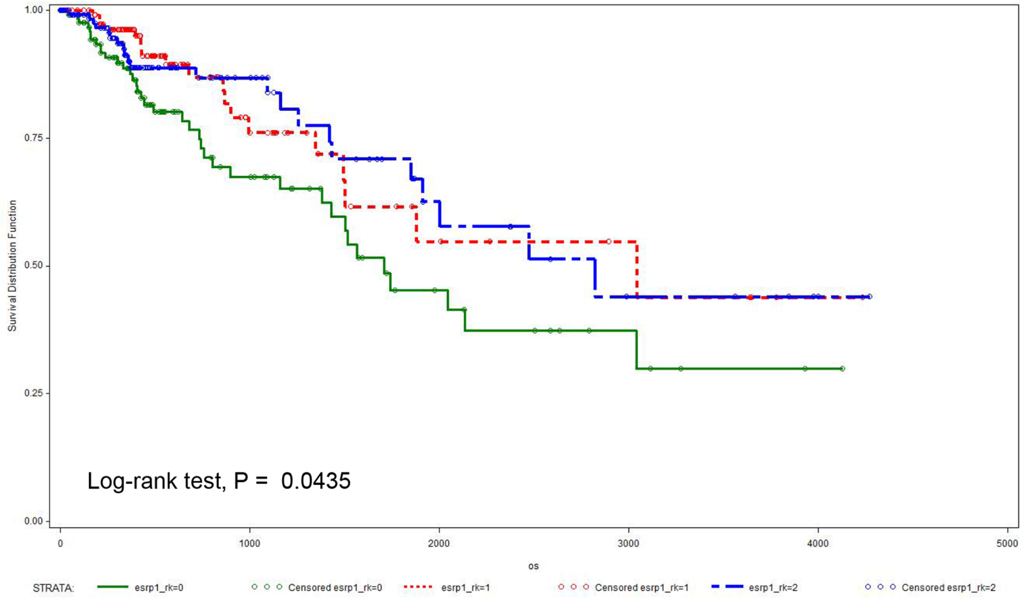

B ESRP2 grouping:
group 0 (-4.96, -0.33, n=138); group 1 (-0.31, 0.465, n=140); group 2 (0.468, 3.00, n=133)

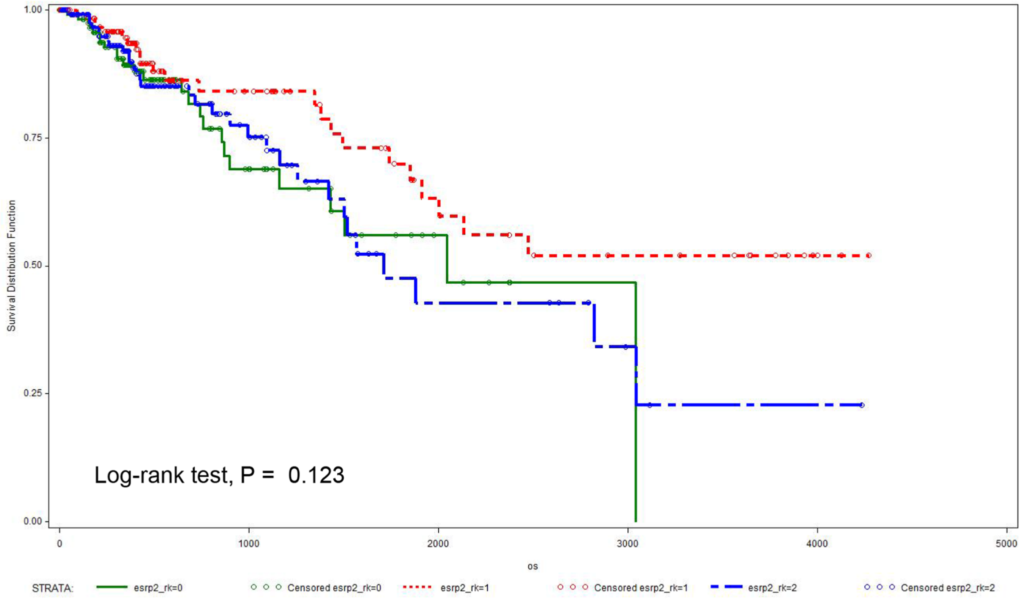

Supplementary Figure S2: Overall survival analyses of CRC patients (n=411) from TCGA COADREAD dataset.

Supplementary Table S1: Correlations of ESRP 1-2 and FGFR 1-3 IIIb/IIIc RQ gene expression levels in tumor (T) and non-tumor (N) tissues of CRC cases*

Tissue	Transcript	ESRP1		ESRP2		
		P-value	coefficient	P-value	coefficient	n
T	ESRP1			**<0.01**	**0.844**	68
	ESRP2	**<0.01**	**0.844**			68
	FGFR1 IIIb/c	0.43		0.19		28
	FGFR2 IIIb/c	0.13		0.30		47
	FGFR3 IIIb/c	0.93		0.98		28
N	ESRP1			**<0.01**	**0.978**	67
	ESRP2	**<0.01**	**0.978**			67
	FGFR1 IIIb/c	**0.01**	**0.487**	**0.01**	**0.471**	27
	FGFR2 IIIb/c	**<0.01**	**0.480**	**0.01**	**0.367**	48
	FGFR3 IIIb/c	0.61		0.59		27

*Pearson correlation coefficients and P-values <0.05 are indicated in bold,
n matched log10 transformed RQ gene transcript level values used for correlation analyses.

Supplementary Table S2: Primer sequences and results of primer pair efficiency rate measurements used for RT-qPCR

Primer*	Sequence (5' … 3')	% Efficiency (R^2)
ESRP1 for	TCCTGCTGTTCTGGAAAGTCG	94.4 (0.999)
ESRP1 rev	TCCGGTCTAACTAGCACTTCGTG	
ESRP2 for	GGGTCTGGGAAGTCAAGACAATG	91.0 (0.998)
ESRP2 rev	CTTCGAAAACAATTGACTGCTGG	
36B4 for	CCCATTCTATCATCAACGGGTACAA	94.0 (0.987)
36B4 rev	CAGCAAGTGGGAAGGTGTAATCC	

*ESRP forward (for) and reverse (rev) primer sequences were obtained from Gemmill et al., 2011 (Cancer Lett, 300, 66-78), 36B4 from Cawthon, 2002 (Nucleic Acids Res, 30, e47).

Supplementary Table S3: Primer sequences and predicted alternative splice product size used for semi-quantitative PCR assays.

See Supplementary File 1
