# Supplementary material for: Epithelial splicing regulatory protein 1 and 2 paralogues correlate with splice signatures and favorable outcome in human colorectal cancer
Source: Oncotarget. 2016 Sep 16;7(45):73800–16. doi: 10.18632/oncotarget.12070 (PMC5342015; doi:10.18632/oncotarget.12070)
Supplement: Supplementary file 2 [file oncotarget-07-73800-s002.doc]

**Supplement Table 3.** Primer sequences and predicted alternative splice product size used for semi-quantitative PCR assays.

| **Primer** | **Sequence (5’ … 3’)** | **Amplicon size in bp (mRNA variant name: nucleotide accession number)*** |
| --- | --- | --- |
| CD44 for CD44 rev | AGGAGCAGCACTTCAGGAGGTTAC ACTGGGGTGGAATGTGTCTTGGTC | **138** (V4: NM_001001391.1, V8: NM_001202557.1) **330** (VX16: XM_011520488.1) **342** (V6: NM_001202555.1) **534** (V3: NM_001001390.1) **795** (VX11: XM_005253238.2) **1023** (VX10: XM_Q11520485.1) **1026** (VX9: XM_Q11520484.1) **1029** (VX8: XM_011520483.1) **1080** (VX7: XM_011520482.1) |
| CTNND1 for CTNND1 rev | GCTGGATTTGTCTTTCTCAGC CCATCATCTGAGGTCTCCAC | **210** (V13: NM_001085469.1) **282** (V7: NM_001085463.1, V8: NM_001085464.1, V9: NM_001085465.1, V10: NM_001085466.1, V12: NM_001085468.1)  **462** (V14: NM_001206883.1, V15: NM_001206884.1, V17: NM_001206886.1, V18: NM_001206887.1, V19: NM_Q01206888.1)  **690** (V4: NM_001085460.1) **763** (V1: NM_001085458.1, V2: NM_001085459.1, V3: NM_001331.2, V5: NM_001085461.1, V16: NM_001206885.1) |
| ENAH for ENAH rev | TGCTGGCCAGGAGGAGAAGAAT ACTGGGCTGTGATAAGGGTGTGG | **218** (V2: NM_018212.4) **281** (V1: NM_001008493.1) |
| 36B4 | (see suppl. Table 1) | **75** (V1: NM_001002.3, V2: NM_053275.3) |

*Amplicons were identified by Primer-BLAST (NCBI, [www.ncbi.nlm.nih.gov/tools/primer-blast/](http://www.ncbi.nlm.nih.gov/tools/primer-blast/)) and by alignment of genome and transcript reference sequences (Clone Manager 9, Scientific & Educational Software, NC).
